# A new class of antimicrobial molecules derived from kefir, effective against *Pseudomonas aeruginosa* and methicillin resistant *Staphylococcus aureus* (MRSA) strains

**DOI:** 10.1038/s41598-020-73651-7

**Published:** 2020-10-15

**Authors:** Vaniky Duarte Marques, Marcia Regina Franzolin, Sabri Saeed Sanabani, Hugo Vigerelli, Roxane Maria Fontes Piazza, Daniel Carvalho Pimenta, Tiago Venâncio, Irys Viana Neves, Herbert Guimarães de Sousa Silva, Daniella dos Santos Courrol, Lucia Mendonça-Previato, José Osvaldo Previato, Soraia Attie Calil Jorge, Marta de Oliveira Domingos

**Affiliations:** 1 Laboratory of Bacteriology, Instituto Butantan, Avenida Vital Brasil, 1500, São Paulo, SP 05503-900 Brasil; 2 Instituto de Medicina Tropical, Faculdade de Medicina da Universidade de São Paulo, São Paulo, SP Brasil; 3 Laboratório de Bioquímica e Biofísica, Instituto Butantan, São Paulo, SP Brasil; 4 Laboratório de Ressonância Magnética Nuclear, Departamento de Química, Universidade Federal de São Carlos, São Carlos, SP Brasil; 5 Laboratório de Glicobiologia, Instituto de Biofísica Carlos Chagas Filho, Universidade Federal Do Rio de Janeiro, Rio de Janeiro, RJ Brasil; 6 Laboratório de Imunologia Viral Instituto Butantan, São Paulo, SP Brasil; 7 Present Address: Laboratório de Genética, Instituto Butantan, São Paulo, SP Brasil

**Keywords:** Drug discovery, Microbiology

## Abstract

Many studies have linked the antimicrobial properties of kefir with the presence of bacteriocins and organic acids. In the present work, results obtained from bacteriostatic and bactericidal studies, and from RP-HPLC, Mass Spectrometry and proton NMR analysis, show that a sample of milk kefir grains is able to produce an antimicrobial fraction, denoted FK-1000, composed of sugars and amino acids, predominantly polymers of alanine, doublets of tyrosine and phenylalanine. Since this fraction is a lyophilized product whose molecular profile is different from bacteriocins and simple carboxylic acids, its antimicrobial effect cannot be attributed to these molecules, or to alcohols or hydrogen peroxide. The fraction is bactericidal against weak-acid-resistant MRSA and weak-acid resistant *P. aeruginosa* at pH 5, and is bacteriostatic against both pathogens at pH 7. In combination formulation, the FK-1000 fraction is able to increase fivefold the effect of streptomycin against *P. aeruginosa* and it is not toxic to human epithelial cells at antimicrobial concentrations. 16 S rRNA microbiota analysis of antimicrobial-producing and non-producing kefir grains demonstrated that they are distinct. In summary, the results indicate that milk kefir grains can produce different classes of molecules with potent antibiotic activity against resistant bacteria.

## Introduction

Traditional Kefir is a fermented milk beverage that has been consumed as an aliment for more than 2000 years^1^. It is produced by a symbiotic association of yeast, lactic acid bacteria (LAB), and/or acetic acid bacteria which are sheltered in an irregularly shaped gelatinous matrix of polysaccharide structures that resemble a cauliflower, and which have been given the name of kefiran^1,2^. Several studies have demonstrated that kefir has antimicrobial activity against a large number of pathogens^3,4^. Its antimicrobial effects, however, have mostly been associated with the presence of antimicrobial peptides and secondary metabolites such as ethanol, acids and acetaldehyde^3,5–10^. Other studies have demonstrated that the antimicrobial activity of kefir varies independent of the presence of these metabolites, but that it is strongly dependent on the source of the grains, which are composed of different species of microorganism^3^.

The microbiological composition of the grains is the subject of much controversy, the composition differing according to the source, which directly influences the type of the metabolites present in kefir^11^. In recent times, these variations are even greater since fruit juices and molasses have been used as substrates to prepare kefir^12,13^. Accordingly, several groups have decided to isolate bacterial strains from the grains and study their ability to produce antimicrobial molecules separately from the whole microbiome^7,14^. The results obtained from those studies demonstrated that several isolates were able to produce bacteriocins, which are antimicrobial molecules genetically coded, synthesized through the ribosomal machinery and classified according to their size, structure and resistance to heat^7,15,16^.

Considering the importance of the intestinal microbiome in maintaining good health, interest has increased in the ability of kefir to help the recovery of normal flora in cases of dysbiosis after antimicrobial treatments^4^.

In light of the high degree of variability found in the metabolites encountered in kefir grains, this work investigated whether some samples of kefir contain molecules different from bacteriocins, acids, alcohols or hydrogen peroxide, with the potential to be developed as commercial antimicrobial products. The results obtained in this study indicate that some batches of kefir grains are indeed able to produce such molecules.

## Methods

### Bacterial samples

The bacteria strains employed in this study were the *Pseudomonas aeruginosa* ATCC-27853 and a Methicillin Resistant *Staphylococcus aureus* (SISGEN A08B202) obtained from the bacterial collection of the Bacteriology Laboratory of the Butantan Institute. Their identification was confirmed by commercially available biochemical identification Systems (API-20NE and API-STAPH-BioMerieux, Marcy-l’Étoile, France) respectively.

A cefoxitin disk was used to define MRSA according to CLSI recommendations^17^. The results confirmed that the *S. aureus* (SISGEN A08B202) is a MRSA strain.

### Kefir grains: sources and procedures

**Figure 1:**
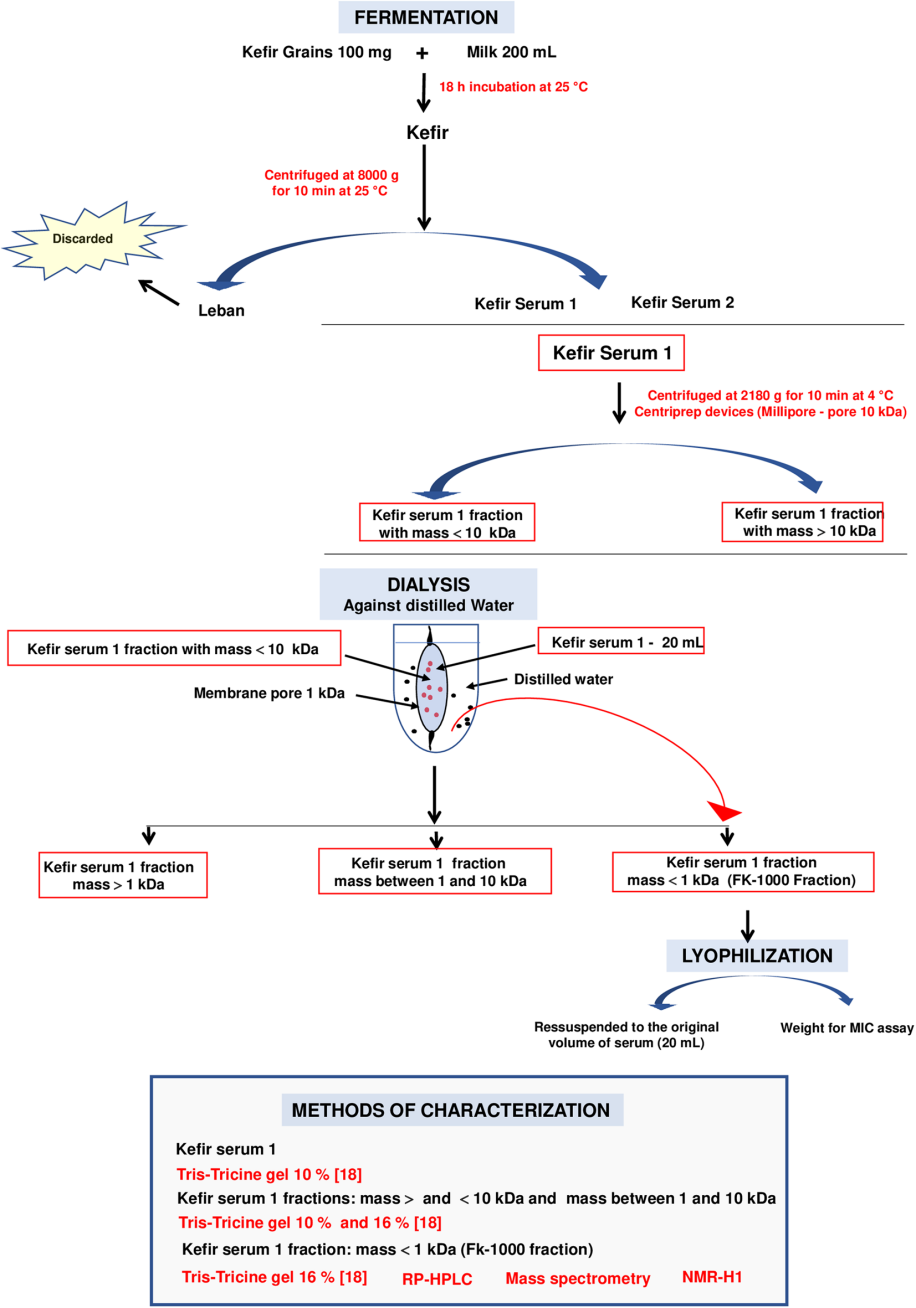
Preparation and analysis of kefir, kefir serum and kefir serum molecular fractions.

Two samples of milk kefir grains were utilized in this study. The kefir grain samples were obtained by donation acquired from different domestic sources (São Paulo-Brazil). The kefir serum produced by the grains were denoted kefir serum 1 and kefir serum 2 and their antibacterial effect were analyzed. Since the results demonstrated that just one of grain samples produced kefir serum with antimicrobial effect (kefir serum 1), this serum was further separated into different molecular fractions. The procedures utilized to obtain kefir, kefir serum, kefir serum molecular mass fractions and their analysis are described graphically in item 2.3 (Fig. 1). The procedures described herein are based on a standard operating system established after recording and repeating several experiments whose results were reproduced consistently.

### Preparation and analysis of kefir, kefir serum and its respective molecular fractions

Two samples of kefir derived from different batches of milk kefir grains (Kefir 1 and Kefir 2) have been prepared with whole pasteurized cow’s milk obtained from a commercial Brazilian supplier, inspected by the Brazilian Agriculture Ministry under registration SIF 2949. The standard operating procedures employed is described in Fig. 1.

### Characterization of the FK-1000 kefir serum antimicrobial molecular fraction

The antimicrobial molecular fraction of kefir serum 1 with mass lower than 1 kDa denoted FK-1000 was characterized by mass spectrometric analysis in conjunction with the HPLC chromatography system (20 A Prominence, Shimadzu Co., Japan) using a C18 column (Discovery 50 × 2.1 mm), with solvents (A) acetyl acid/H_2_O (1:1000) and (B) acetic acid/acetonitrile/H_2_O. The gradient used was from 0 to 100% of solvent B over 20 min. A 4.5 kV, voltage detector was employed 1.76 kV at a temperature of 200 °C, 50–2000 m/z. Data was analysed using the LCMS solution software (Shimadzu Co., Japan). The fraction was also analysed by proton NMR in a 1-D Equipment using D20 as solvent (deuterated water) and measuring the 1H signal at a proton frequency of 500 MHz.

The data obtained from mass spectrometric and RP-HPLC analysis were used to define a molecular signature for the antimicrobial FK-1000 fraction.

### Identification of the microbiota present in the milk kefir grains

The microbiota present in the milk kefir grains responsible for the production of kefir serum 1 and kefir serum 2 was analyzed using 16S rRNA gene massive parallel sequencing (MPS) technology for bacterial identification.

Briefly, genomic DNA was extracted from each sample of kefir grains using the PowerSoil DNA kit (MoBio Laboratories, Carlsbad, CA, USA) according to the manufacturer’s instructions. After extraction, the DNA was submitted for amplification of the V3 and V4 region of the 16 S rRNA gene using a two-step polymerase chain reaction (PCR) protocol with the primers Bakt_341F/Bakt_805R^19^. All reactions, purifications, and library preparations were performed according to conditions previously established and described by our group^4,20^.

Image analysis, data base and quality assessment were initially performed on the MiSeq instrument (San Diego, CA, USA). Sequence cleaning and analysis was conducted according to recently published methods^21,22^. The MPS data have been uploaded to Zenodo: https://doi.org/10.5281/zenodo.3560871.

### Antibacterial activity

The capacity of two samples of kefir serum produced by grains from different sources to inhibit the growth or to kill *P. aeruginosa* and MRSA was determined at pH 5 and 7.

The bacteriostatic effect was defined as the ability of the samples to inhibit bacterial proliferation. Briefly, *P. aeruginosa* and MRSA previously grown in Mueller Hinton medium at 37 °C for 18 h (10^4^ cells/ mL) were added to the wells of a 96-well tissue culture plate containing 200 μL/well of Muller Hinton medium either in the presence or absence of each sample of kefir serum. The plate was then incubated for 18 h at 37 °C and bacterial proliferation was determined by reading the plate optical density at 595 nm in a Multiskan EX (LabSystem) reader. The bacteriostatic activity of the samples was calculated according to their ability to inhibit bacterial proliferation. Microbicidal activity was determined by counting the number of colony forming-units (CFU). The same culture plate used for the proliferation assay was used for counting the number of CFUs. Shortly, after reading the optical density of the plates, 100 µL of culture from each well was added to 900 µL of 0.9% saline and subsequently subjected to 25 serial ten-fold dilutions. Then, 10 µL of each dilution was added in triplicate on Tryptic Soy Agar (TSA) plates that were incubated for 18 h at 37 °C and the number of CFU was determined according to the protocol established by Baron et al.^23^ and Beckk et al.^24^. The activity was considered bactericidal when 99.9–99.99% of the pathogens were killed^25^.

The same procedures were used to determine the bacteriostatic and bactericidal effects of the molecular fractions separated from the antimicrobial kefir serum described in “Preparation and analysis of kefir, kefir serum and its respective molecular fractions” (Fig. 1).

Determination of the Minimum Inhibitory Concentration (MIC) of the FK-1000 fraction, was performed according to CLSI guidelines^26^.

### Effect of the FK-1000 fraction in combination with streptomycin

In order to determine whether the FK-1000 fraction could potentiate the antimicrobial effect of an aminoglycoside in the broth, a streptomycin microdilution assay was employed^26^. Briefly, a culture of *P. aeruginosa* previously grown in Tryptic Soy Broth (TSB) for 18 h containing (10^4^ cells/mL) was incubated for 18 h at 37 °C with the following concentrations of streptomycin: 16 µg/mL, 8 µg/mL, 4 µg/mL, 2 µg/mL, 1 µg/mL, 0.5 µg/mL and 0.25 µg/mL (100 µL/well) in the presence or absence of a concentration of 25 mg/mL of the FK-1000 fraction (100 µL/well). After incubation, the bacterial proliferation was determined by reading the optical density of the plate at 595 nm in a Multiskan EX (LabSystem) reader. The experiment was carried out at pH 7 and the results were calculated according to the following equation:

$$\% {\text{Cell}}\;{\text{inhibition}} = \frac{{\left[ {{\text{control}}\;{\text{at}}\;{\text{OD}}_{{595\;{\text{nm}}}} - {\text{test}}\;{\text{at}}\;{\text{OD}}_{{595\;{\text{nm}}}} } \right]}}{{{\text{control}}\;{\text{at}}\;{\text{OD}}_{{595\;{\text{nm}}}} }} \times 100$$

### Cytotoxic effect of the FK-1000 fraction on epithelial cells

The cytotoxic effect of the FK-1000 fraction on human epithelial cells (HEp-2) was determined by the MTT method, which assesses the viability of cells in terms of their mitochondrial metabolic rate. Accordingly, 100 µL of Dulbecco’s Modified Eagle’s Medium (DMEM) containing 10^6^ cells/mL was added to the wells of a 96 well cell culture plate and incubated for 24 h at 37 °C in a 5% CO_2_ incubator. After incubation, the medium was discarded and 100 µL of a concentration of 50 mg/mL of the FK-1000 fraction was added to the plates (100 µL/well) and incubated 18 h at 37 °C in a 5% CO_2_ incubator. After incubation, the supernatant was discarded and 20 µL of a 5% solution of MTT in PBS was then added to each well and the plate was incubated for 2 h at 37 °C. One hundred microliters of Triton X (1%) was used as positive control. Subsequently, 100 µL/well of methanol (100%) was added to the plate and then incubated for a further 10 min. After incubation, the absorbance of each sample was determined at 570 nm in a Multiskan EX (LabSystem) reader. The experiment was performed at pH 7.

### Statistical analysis

Statistical analysis was carried out using GraphPad Prism version 6.0.1 (GraphPad Software, Inc., San Diego, CA, USA) employing the unpaired, two-tailed Student’s *t* test. *p*-values less than 0.05 are considered statistically significant.

## Results

### Antimicrobial activity of two samples of kefir serum produced from grains of different sources

In order to determine the influence of low and neutral pH on the antibacterial activity of two samples of kefir serum (1 and 2) produced by grains derived from different sources, their pH was adjusted to 5 and 7 and their bacteriostatic and bactericidal effects were determined against weak-acid-resistant *P. aeruginosa* and MRSA strains.

**Figure 2:**
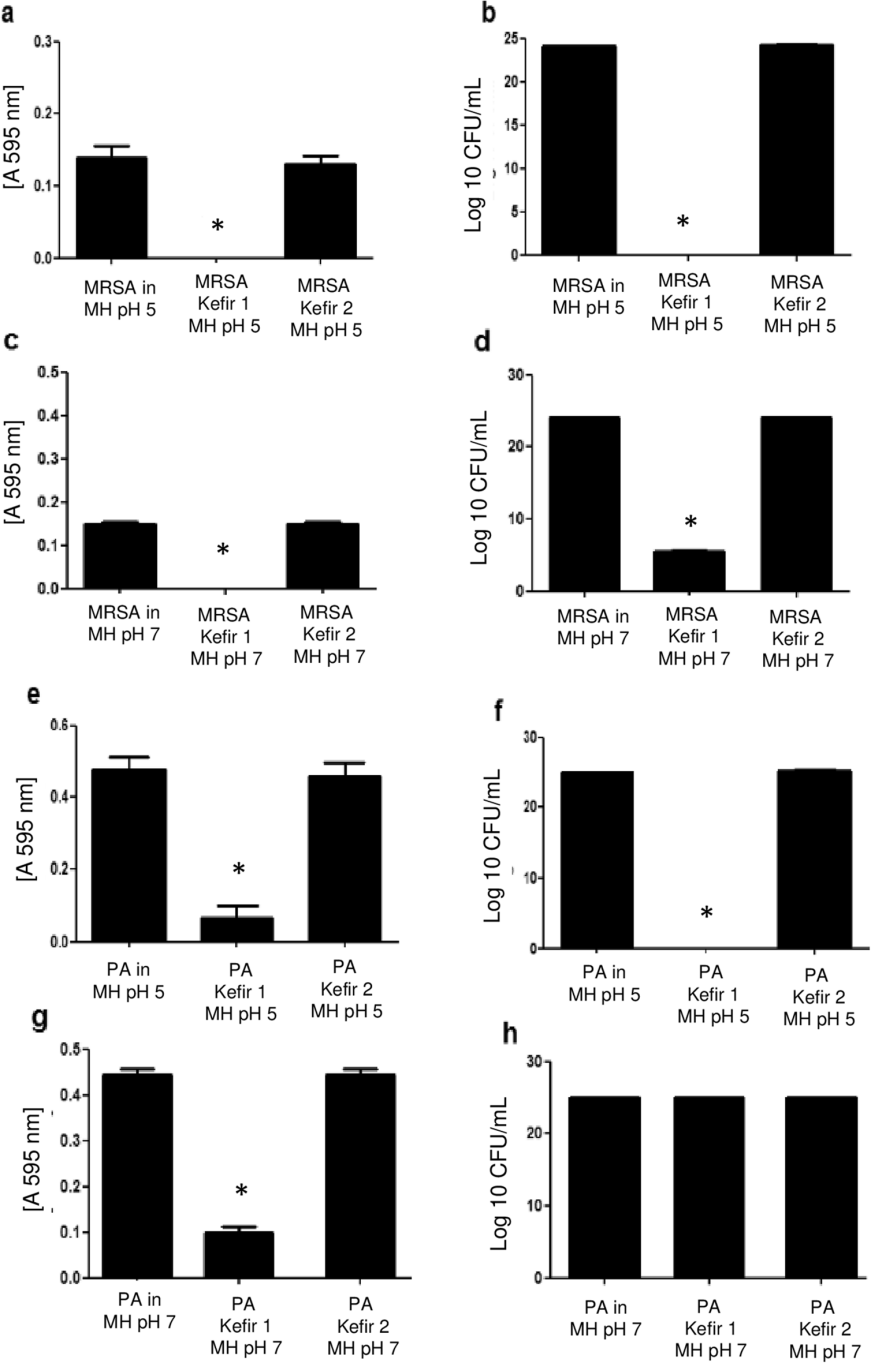
Antimicrobial activity of kefir 1 and kefir 2 serum samples. The antimicrobial properties of kefir serum produced by grains derived from different sources were evaluated against MRSA and *P. aeruginosa* (PA) strains at pH 5 and pH 7. MRSA and PA cultures were incubated with Mueller Hinton medium (MH) either in the presence or absence of two different samples of kefir serum denoted kefir 1 and kefir 2. After 18 h incubation at 37 °C, proliferation (**a**, **c**, **e**, **g**) and CFU (**b**, **d**, **f**, **h**) were measured to determine their bacteriostatic and bactericidal effects respectively. *Statistically significant (p ≤ 0.05) difference between experimental and control (bacteria incubated only with MH) groups.

The results demonstrated that the kefir 1 sample at pH 5 (Fig. 2a,b) and pH 7 (Fig. 2c,d) has bactericidal activity against MRSA strain. In contrast, only at pH 5 did the kefir serum 1 show any bactericidal effect against *P. aeruginosa* (Fig. 2e,f), although at pH 7 it had a bacteriostatic effect (Fig. 2g,h). The kefir 2 sample, however, did not show any antimicrobial effect against *P. aeruginosa* or MRSA at either pH (Fig. 2).

### Characterization of the bacterial microbiota of two different sources of milk kefir grains

**Figure 3:**
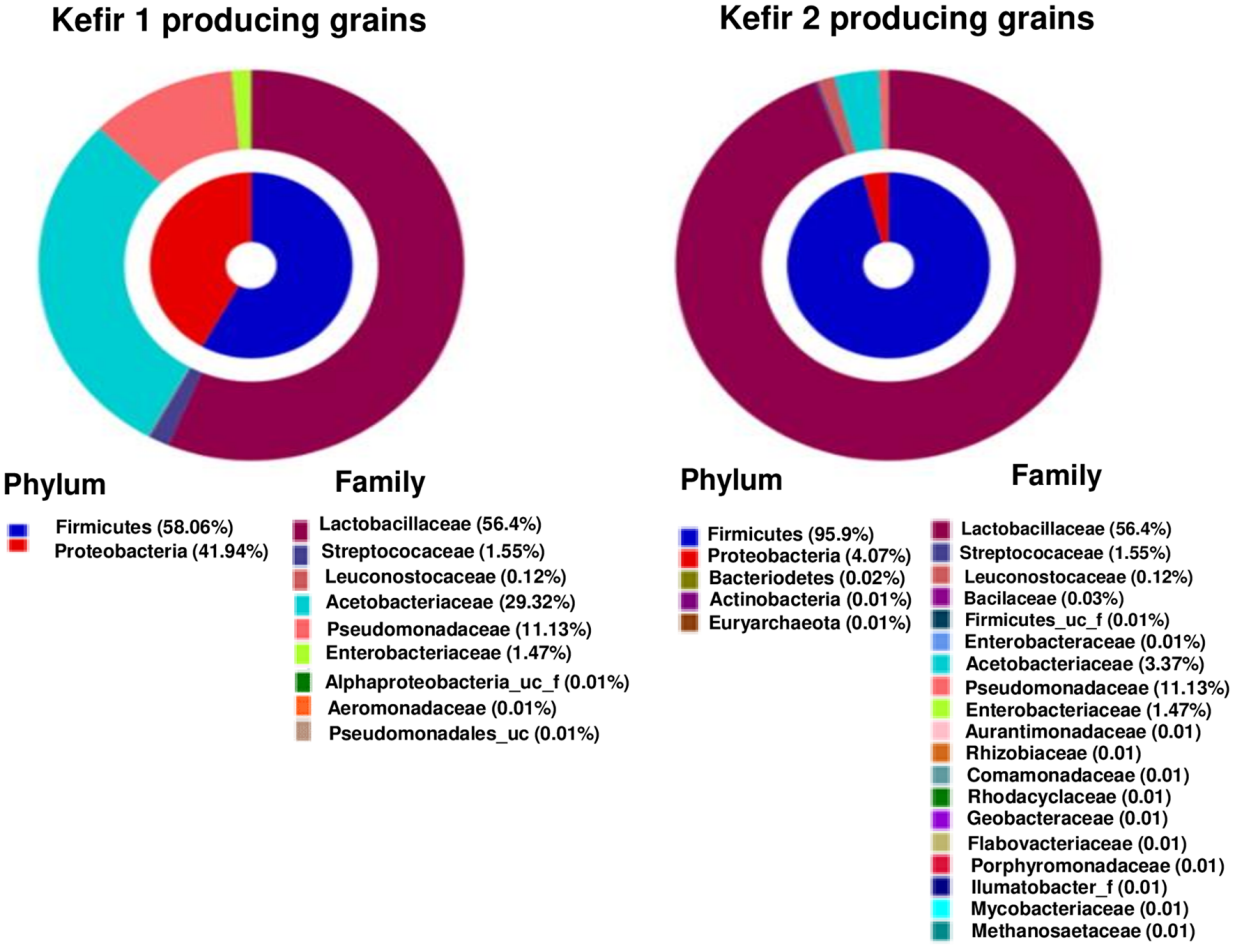
16S rRNA bacterial microbiota characterization of the milk kefir grain samples responsible for the production of kefir 1 and kefir 2.

Since kefir serum 1 demonstrated antibacterial activity against *P. aeruginosa* and MRSA strains whereas kefir serum 2 had no antibacterial activity against these pathogens, it was decided to determine whether the microbiota present in the grains that produced kefir 1 and the grains that produced kefir 2 differed significantly. The results obtained by 16S rRNA analysis showed the presence of nine different families of bacteria in the grains that produced kefir 1 with antimicrobial activity. In contrast, 19 bacterial families were present in the grains that produced kefir 2 with no antimicrobial activity. The main families present in the kefir 1 grains were *Lactobacilaceae* (56.4%), *Acetobacteraceae* (29.32%), *Pseudomonadacea* (11.13%) and *Streptococcaceae* (1.55%) (Fig. 3). In contrast, the vast majority of bacterial species present in the kefir 2 grains belonged to the *Lactobacilaceae* family (94.43%). Furthermore, the prevalence of the other 18 bacterial families present in the grains that produced kefir 2 was very low (Fig. 3).

From here on, only the results obtained with the antibacterial kefir serum 1 are presented.

### Characterization of the antimicrobial kefir serum and its constituent fractions

**Figure 4:**
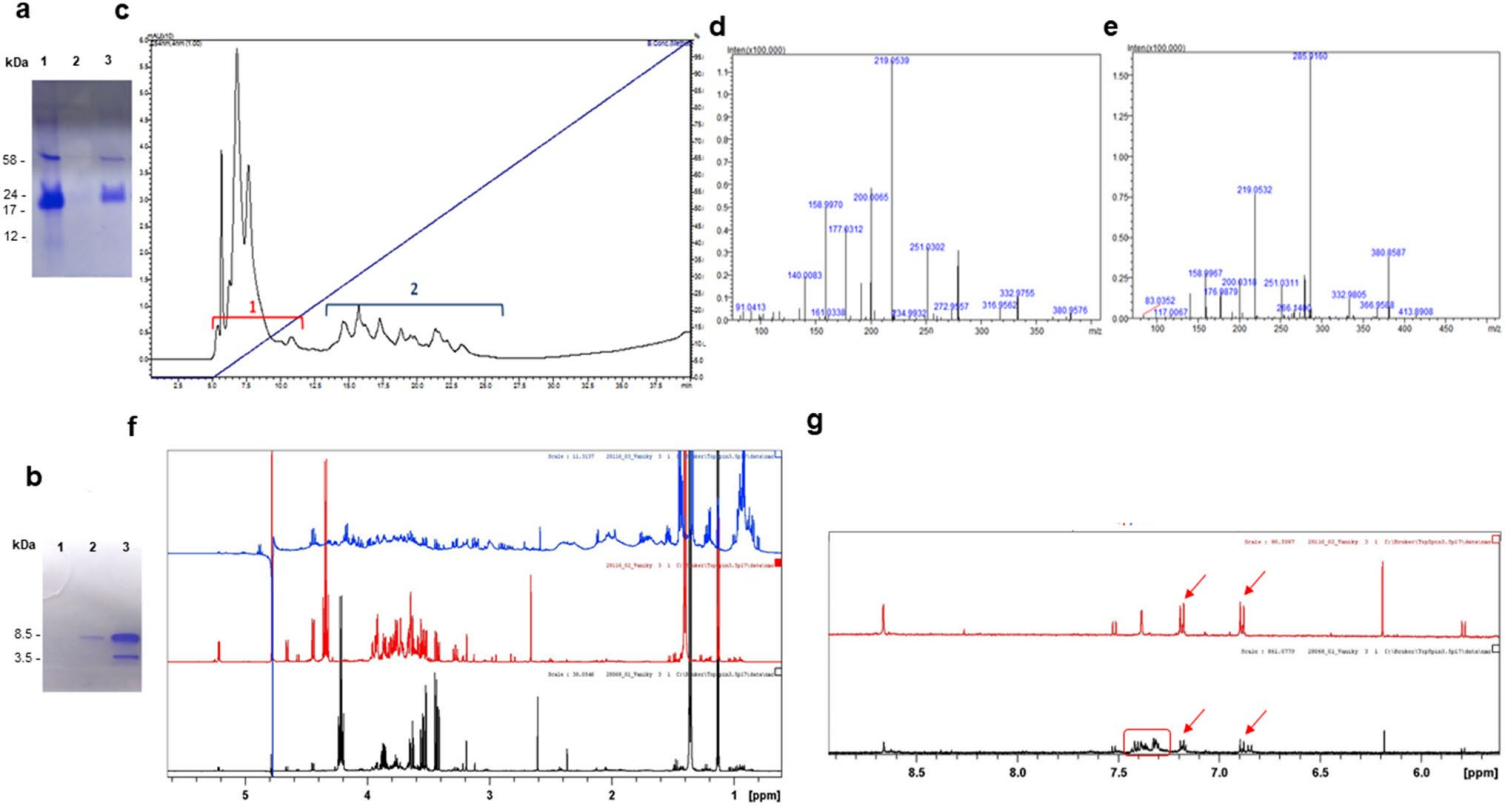
Characterization of the antimicrobial Kefir 1 serum and its molecular fractions. (**a**) Tris-Tricine 10% gel 1—Kefir serum, 2—Kefir molecular fraction with mass > than 10 kDa (diluted 1/10), 3—Kefir molecular fraction with mass > than 10 kDa (diluted 1/2); (**b**) Tris-Tricine gel 16% 1—KF 1000 fraction, 2—molecular fraction with mass between 1 and 10 kDa (diluted 1/10), 3—molecular fraction with mass between 1 and 10 kDa (diluted 1/2). The full-length Tris-Tricine gels are presented in Supplementary Figure S1. (**c**) RP HPLC profile in C18 of the FK-1000 fraction, indicating the two collected sub-fractions; (**d**) mass spectrometry of the RP HPLC sub-fraction 1; (**e**) mass spectrometry of the RP HPLC sub-fraction 2; (**f**) proton NMR—aliphatic region: RP HPLC sub-fraction 2 (Blue), FK-1000 Fraction (Red), RP HPLC sub-fraction 1 (Black); (**g**) NMR H1—aromatic region: RP HPLC sub-fraction 2 (blue), FK-1000 fraction (Red), RP HPLC sub-fraction 1 (Black). Red arrows—tyrosine, red rectangles—phenylalanine.

The results obtained by Tris-Tricine electrophoretic analysis in 10% gel demonstrate that most molecules present in the kefir serum have a mass between 17 and 31 kDa, with one band above 24 kDa and a faint band below 12 kDa (Fig. 4a), (Supplementary Fig. S1a). The results from Tris-Tricine electrophoresis in a 16% gel show the presence of two bands in the kefir fraction with mass between 1 and 12 kDa. However, no band was observed in the FK-1000 fraction (passing across a 1 kDa cut-off dialysis membrane), confirming that molecules in this fraction have a molecular weight lower than 1 kDa (Fig. 4b), (Supplementary Fig. S1b).

Running of the FK-1000 fraction on RP-HPLC demonstrated the presence of two sub-fractions denoted 1 and 2 (Fig. 4c). Results obtained by mass spectrometry indicated that the molecular mass of the components of the FK-1000 fraction and its respective RP HPLC sub-fractions 1 and 2 ranged from 91 to 800 Daltons (Fig. 4d,e). The analysis of the FK-1000 fraction by NMR H1 showed that the FK-1000 fraction is composed mainly of sugars (β-glucose, α-glucose and β-galactose), and aliphatic amino acids, predominantly polymers of alanine, tyrosine and phenylalanine (Fig. 4f,g).

H1 NMR analysis of the sub-fraction 1 demonstrated a similar profile to the FK-1000 fraction (Fig. 4f), however, with the predominance of sugar and alanine polymers. In contrast, the proton NMR results of the sub-fraction 2 indicated the presence of a very complex mixture that was impossible to be characterized (Fig. 4f). Nevertheless, in the aromatic region, phenylalanine was detected in the FK-1000 fractions and its RP-HPLC sub-fractions 1 and 2 (Fig. 4g).

**Figure 5:**
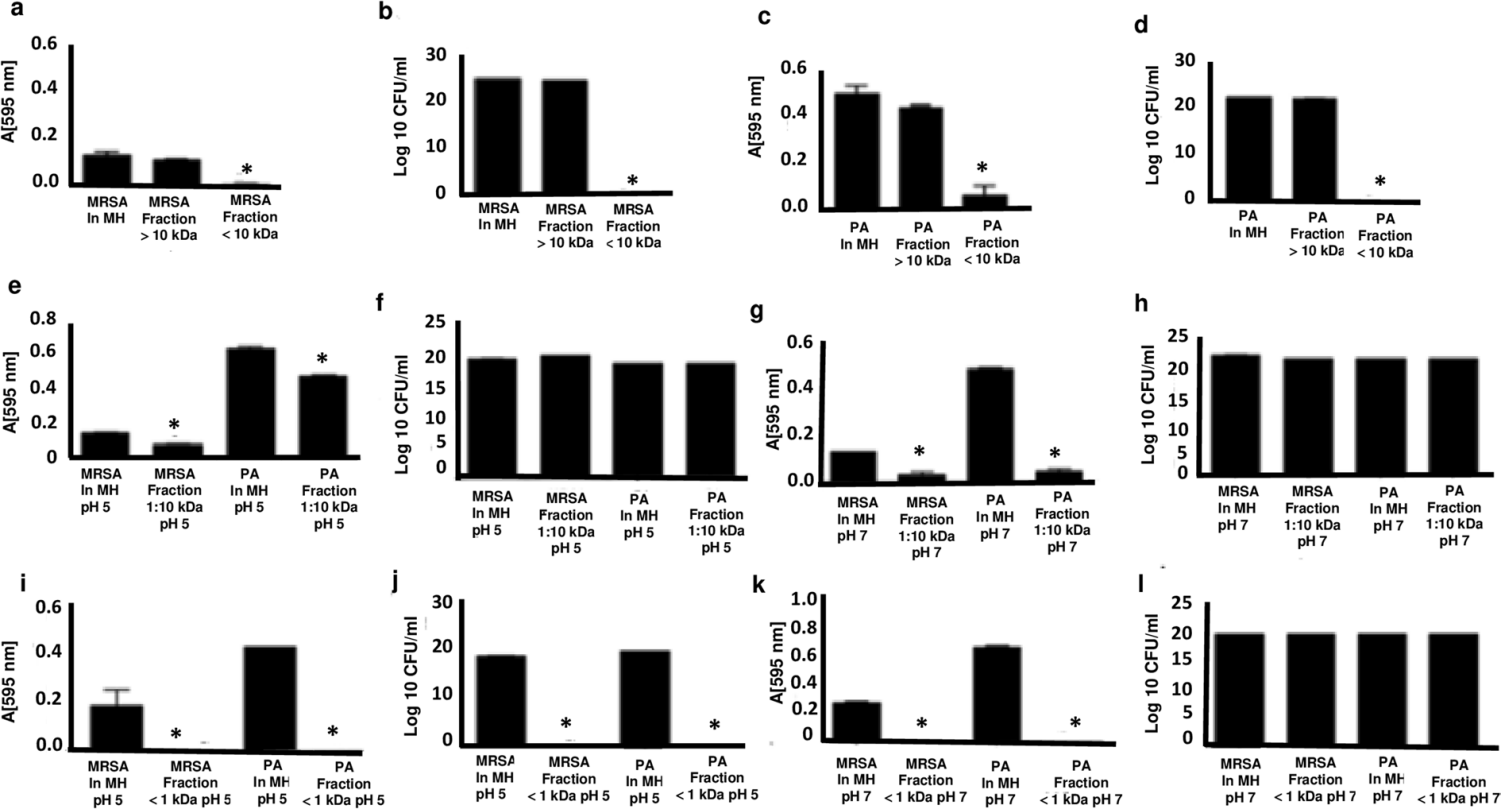
Antimicrobial effect of the kefir 1 fractions. Mass higher and lower than10 kDa at pH 5 (**a**–**d**); mass between 1 and 10 kDa at pH 5 (**e**, **f**); mass between 1 and 10 kDa at pH 7 (**g**, **h**); mass lower than 1 kDa at pH 5 (**i**, **j**) and mass lower than 1 kDa at pH 7 (**k**, **l**). The antimicrobial effect of the fractions were determined against MRSA and *P. aeruginosa* (PA). Accordingly, the MRSA and PA cultures were incubated with Mueller Hinton medium (MH) either in the presence or absence of the molecular fractions. After 18 h incubation at 37 °C, the proliferation (**a**, **c**, **e**, **g**, **i**, **k**) and the CFU (**b**, **d**, **f**, **h**, **j**, **l**) were quantified in order to determine their bacteriostatic and bactericidal effects respectively. *Statistically significant (p ≤ 0.05) difference between experimental and control (bacteria incubated only with MH) groups.

The results observed with kefir molecular fractions with mass higher and lower than 10 kDa demonstrated that the antimicrobial components of kefir have mass lower than 10 kDa (Fig. 5a–d).

The results of the fraction with mass between 1 and 10 kDa demonstrated that the molecules present in this fraction have only bacteriostatic effects against *P. aeruginosa* and MRSA strains at pH 5 and 7 (Fig. 5e–h). The same result was obtained with the molecular fraction with mass higher than 1 kDa (data not shown).

On the other hand, the FK-1000 fraction whose mass is lower than 1 kDa, demonstrated a bactericidal effect against *P. aeruginosa* and MRSA at pH 5, but not at pH 7 (Fig. 5i,j), although it showed bacteriostatic effects against both pathogens at pH 7 (Fig. 5k,l).

**Figure 6:**
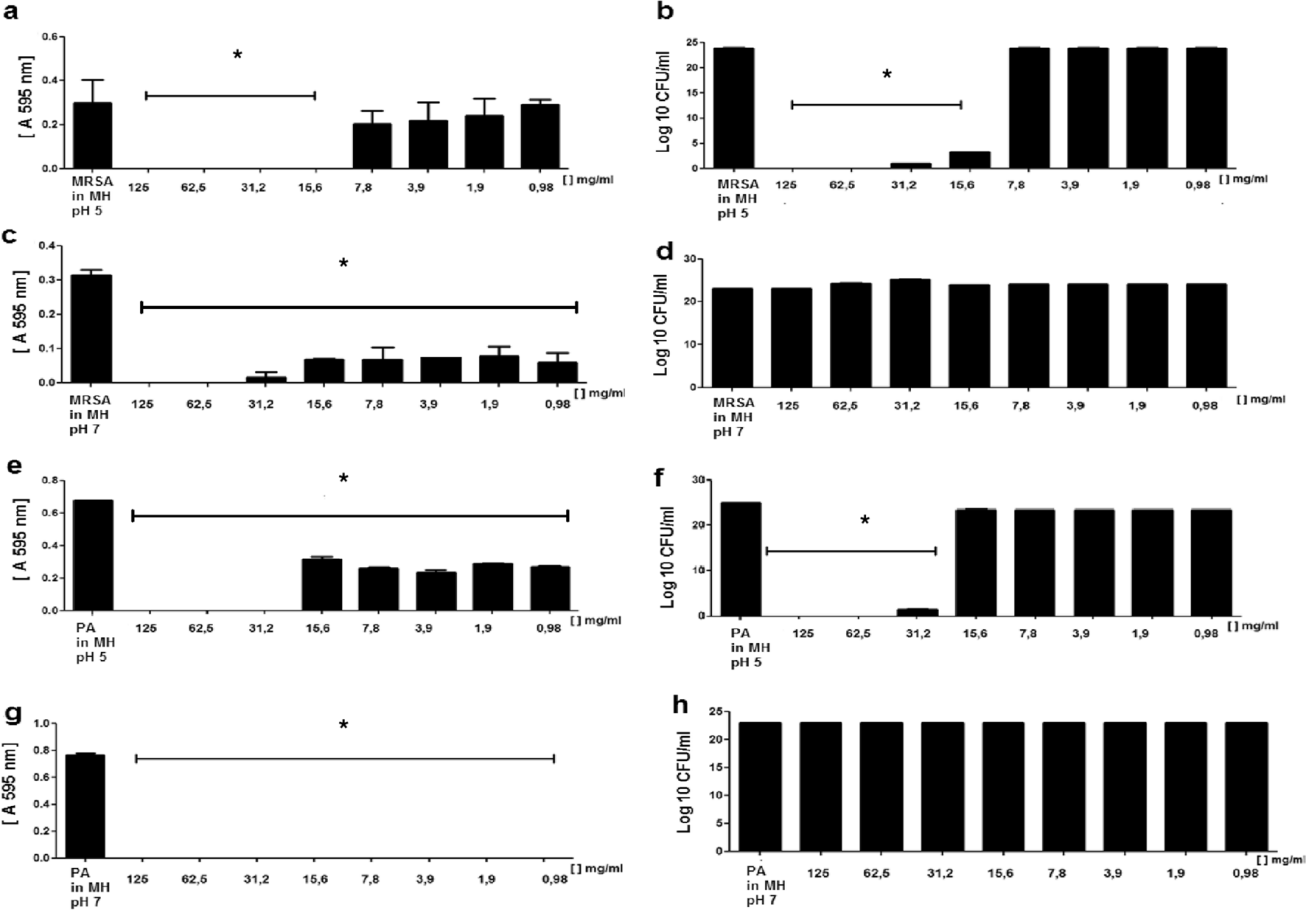
Minimum inhibitory concentration of the FK-1000 fraction against MRSA and *P. aeruginosa* (PA). MRSA and PA cultures in Mueller Hinton medium (MH) were incubated in the presence or absence of different concentrations of the FK-1000 fraction at pH 5 and pH 7. After 18 h incubation at 37 °C proliferation (**a**, **c**, **e**, **g**) and the CFU (**b**, **d**, **f**, **h**) were determined in order to evaluate the bacteriostatic and bactericidal effects of the fraction respectively. *Statistically significant (p ≤ 0.05) difference between experimental and control (bacteria incubated only with MH) groups.

The results have also demonstrated that at pH 5, the minimum concentration of the FK-1000 fraction necessary to induce a bactericidal effect is 15 mg/mL against MRSA and 31.2 mg/mL to *P. aeruginosa* (Fig. 6a–d).

In contrast, at pH 7, despite being able to inhibit the proliferation of both pathogens at a concentration as low as 0.98 mg/mL, the FK-1000 fraction had no bactericidal effect against these pathogens even at the highest concentration of 125 mg/mL (Fig. 6e–h).

**Figure 7:**
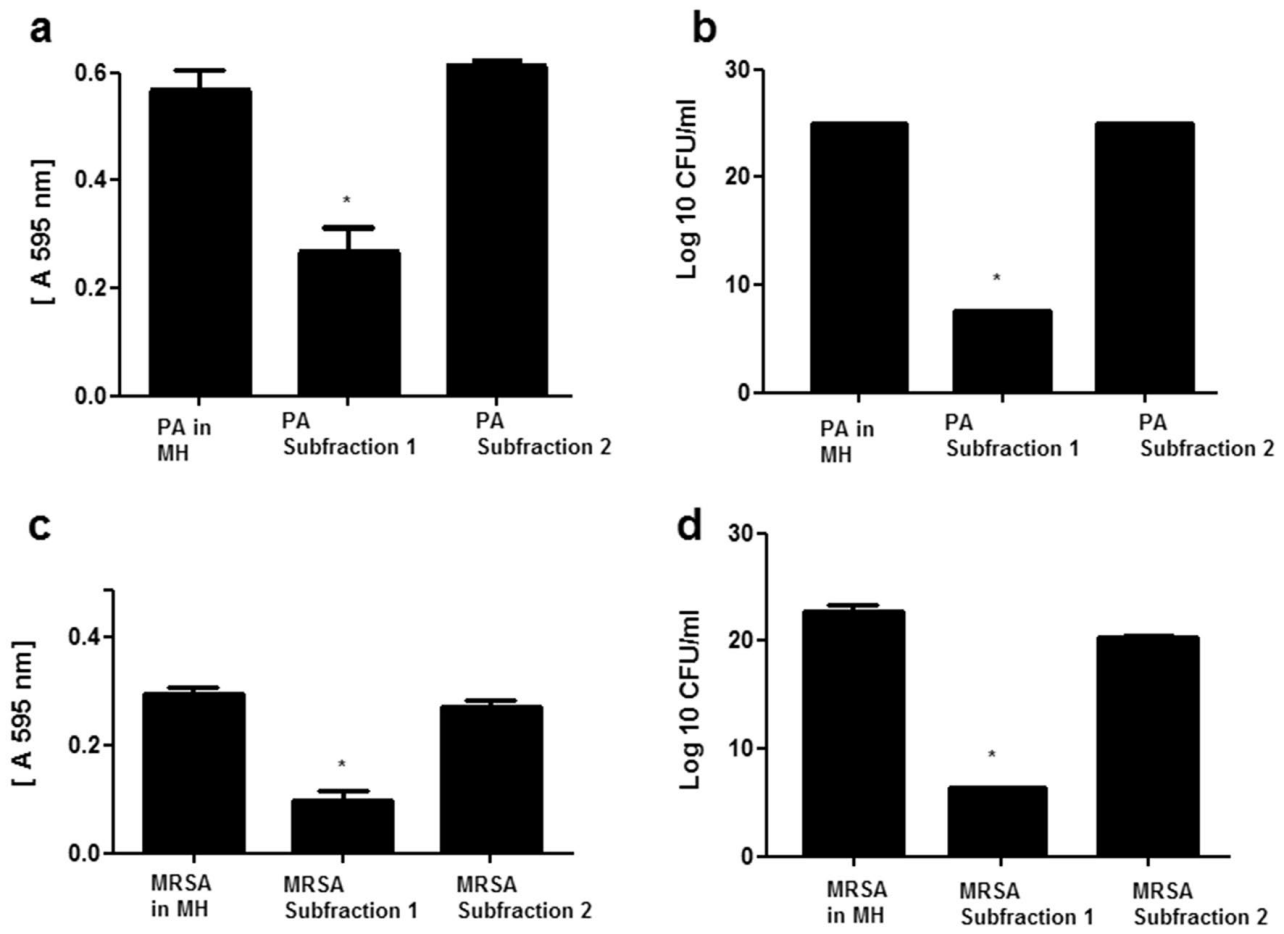
Antimicrobial effect of the FK-1000 RP HPLC sub-fractions. The antimicrobial effect of the RP HPLC sub-fraction 1 and the RP HPLC sub-fraction 2 were determined against MRSA and *P. aeruginosa* (PA) at pH 5. The MRSA and PA cultures were incubated with Mueller Hinton medium (MH) either in the presence or absence of the sub-fractions. After 18 h incubation at 37 °C, the proliferation (**a**, **c**) and the CFU (**b**, **d**) were quantified in order to determine the bacteriostatic and bactericidal activities respectively of the fractions. *Statistically significant (p ≤ 0.05) difference between experimental and control (bacteria incubated only with MH) groups.

Since the antimicrobial effect of the FK-1000 fraction against *P. aeruginosa* and MRSA was better achieved at pH 5, the bacteriostatic and bactericidal effect of the RP HPLC sub-fractions 1 and 2 were tested just at pH 5. The results demonstrated that the antibacterial activity was found only in the sub-fraction 1, whereas the sub-fraction 2 showed no antimicrobial effect against either pathogens (Fig. 7).

### Effect of the FK-1000 fraction in combination with streptomycin

**Figure 8:**
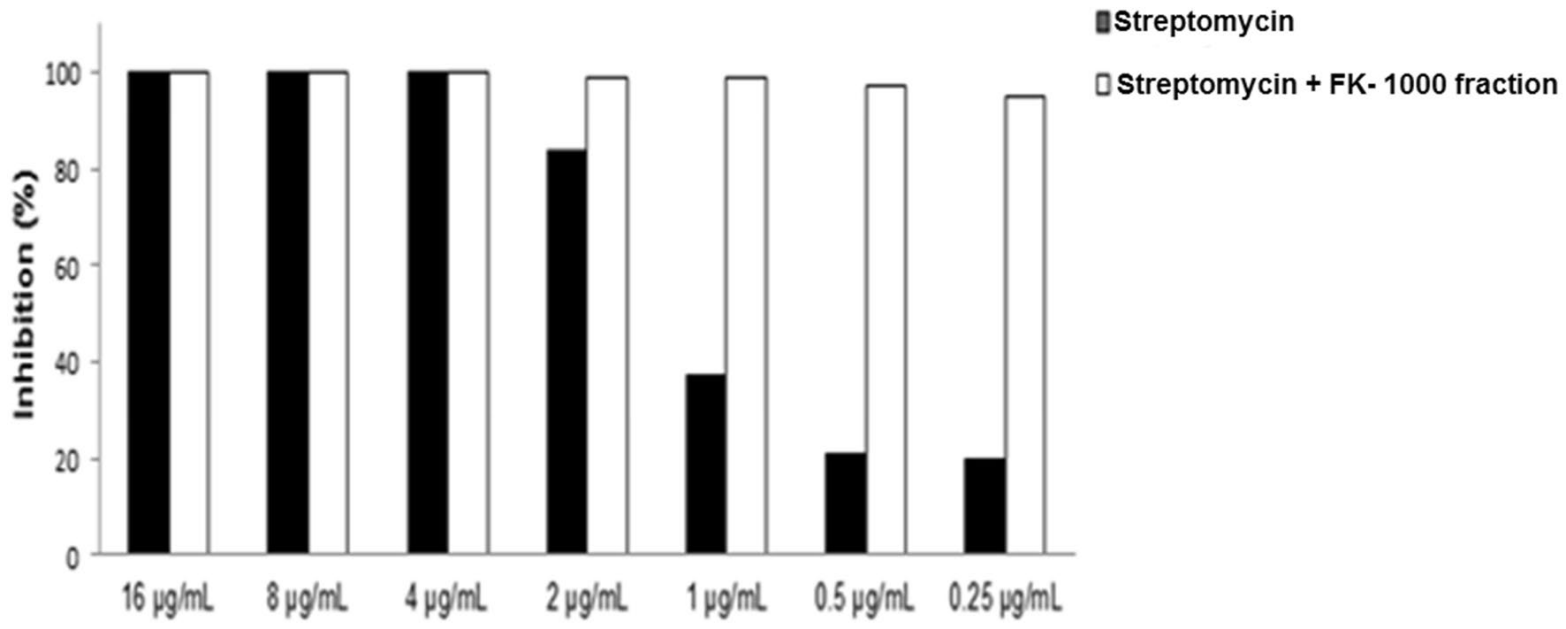
Effect of the formulation composed by the FK-1000 fraction with an aminoglycoside. *P. aeruginosa* culture was incubated in Mueller Hinton medium with different concentrations of streptomycin in the presence or absence of 25 mg of the FK-1000 fraction. After 18 h incubation at 37 °C the percentage inhibition of bacterial proliferation was determined by reading the optical density of the cultures at 595 nm.

In order to determine whether the FK-1000 fraction could act in combination with a broad spectrum aminoglycoside, *P. aeruginosa* was incubated with different concentrations of streptomycin in the presence or absence of the FK-1000 fraction. The results indicated that the FK-1000 fraction increased by almost fivefold the antimicrobial effect against *P. aeruginosa* in combination therapy, since streptomycin alone at 0.25 µg/mL inhibited 19.6% of bacterial proliferation, however, in the presence of the FK-1000 fraction the same concentration of steptomycin was able to inhibit bacterial proliferation at 95.3% (Fig. 8). It is important to note that the concentration of the FK-1000 fraction utilized in this experiment (25 mg/mL) was able on its own to inhibit 91% of bacterial growth.

### Cytotoxic effect of the FK-1000 fraction

**Figure 9:**
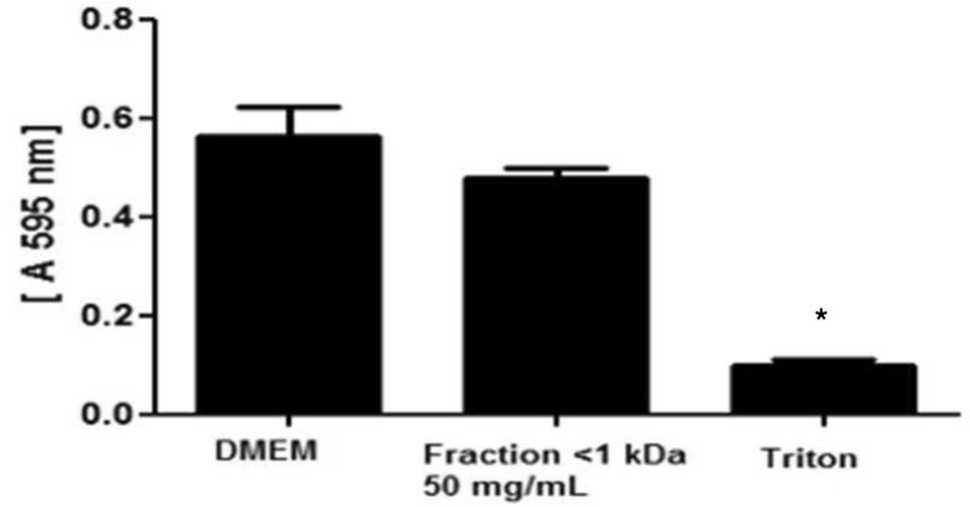
Effect of the FK-1000 fraction on human epithelial cell viability. HEp-2 cells (10^6^/mL) were seeded in a 96 well cell culture plate (100 µL/well) and incubated at 37 °C in a CO_2_ chamber for 18 h with 50 mg/mL (100 µL/well) of the FK-1000 fraction in DMEM. After incubation, the toxicity was determined by the MTT method. Triton (1%) was used as positive control. *Statistically significant (p < 0.05) difference between experimental and control (cells incubated only with DMEM) groups.

Since 50 mg/mL of the FK-1000 fraction is higher than the minimum concentration necessary to induce bactericidal effect against the *P. aeruginosa* and MRSA strains, the MTT assay was used to determine the cytotoxic effect of 50 mg/mL of the FK-1000 fraction on epithelial cells. The results demonstrated that at this concentration the FK-1000 fraction is not toxic to human epithelial cells (Fig. 9).

## Discussion

The present work has demonstrated that the antibacterial activity of kefir against MRSA and *P. aeruginosa* strains is strongly associated with the source and the composition of the kefir grains in terms of the microorganisms present, since the antimicrobial-producing and the antimicrobial-nonproducing grains have distinctly different microbiota. Despite the presence of 19 different bacterial families in the antimicrobial nonfunctional grains, 94.43% of all bacterial species encountered belonged to the *Lactobacilaceae* family, which according to the scientific literature has a high number of bacterial species able to produce antimicrobial peptides such as bacteriocins^7,14,27^. In contrast, only nine bacterial families were encountered in the antimicrobial-producing grains, the *Lactobacilacea* family being responsible for 56% of them. However, the possibility cannot be discounted that other microorganisms such as yeasts present in the microbiome of the grains could also be responsible for the capacity of kefir to kill bacteria.

All Kefir serum samples were prepared with the same source of milk and in the exact same manner, so the antibacterial activity encountered cannot be associated with the direct action of the milk on the MRSA and *P. aeruginosa* strains, but rather by milk-derived components generated by the action of the enzymes produced by the microbiome organisms present in the grains during fermentation^28,29^.

The MRSA and *P. aeruginosa* strains utilized in this study were acid resistant and could normally grow at pH 4.5, and the kefir serum sample had microbicidal effects against the MRSA strain even at pH 7. This is an important result to note, since antimicrobial molecules often react differently at different pHs^30,31^. Thus, the power of kefir serum to kill bacteria is attributable not to a low pH but to antimicrobial molecules produced during fermentation, in corroboration with other studies that demonstrated that the antimicrobial activity of four different samples of kefir was not related to their low pH (between 3.65 and 4)^3^.

The fact that the kefir serum showed antimicrobial effect against *P. aeruginosa* and MRSA strains is a very important finding, since these bacteria, along with *Enterococcus faecium*, *Klebsiella pneumoniae*, *Acinetobacter baumanni* and *Enterobacter* spp., are responsible for most cases of nosocomial infections worldwide^32^.

However, in light of the fact that the capacity of unpurified kefir serum to kill bactteria was different from that observed for its molecular fractions, certain points need to be considered. For instance, the unpurified kefir serum was bactericidal against MRSA at acid and neutral pH, whereas the FK-1000 fraction was bactericidal against this pathogen only at pH 5. In contrast, the molecular fraction of kefir serum with mass between 1 and 10 kDa demonstrated only bacteriostatic effect. This result is in accord with studies that demonstrated that the majority of bacteriocins produced by bacterial strains isolated from kefir and traditional fermented milk, have mass between 1 and 10 kDa^7,16,33,34^.

Altogether these results indicate that the combined effect of the antimicrobial molecules within kefir can be different from the effects of those seen when isolated from it. This may be because the antibacterial activity of unpurified kefir serum results from the synergy of several molecules, so their separation could lead to a different and/or weaker antimicrobial response.

One of the ways in which antibiotics combat pathogenic bacteria is by interfering in the regulation of the enzymes involved in the cell wall biosynthesis^35,36^. They can use two different approaches—targeting either their peptidoglycan glycosyltransferases (GT) or their peptidoglycan transpeptidases (PT) which are enzymes essential to the integrity of the bacterial cell wall^35,36^.

A good example of an antibiotic that targets GT is moenomycin, which is a pentasaccharide that mimics the real substrate of GT (disaccharide-pyrophosphate-prenol)^36^. Synthesized monosaccharides with moenomycin-like activity such as ACL20965 and ACL 29215 were also able to clear in vivo 99% of Staphylococci infected murine mammary glands^36^.

Another class of antibiotic that also acts on the bacterial cell wall is that of β-lactams such as penicillin. In this case they target peptidoglycan transpeptidases (PT) using sequences of alanine (d-Ala-d-Ala) that mimic the natural substrate (d-Alanyl-d-alanine) of peptidoglycan transpeptidases^37,38^. Therefore, by inhibiting the activity of these transpeptidases, the d-alanine sequences present in the β-lactams impair the formation of the bacterial cell wall, consequently inducing bacterial death.

In addition, it has been demonstrated that residues of phenylalanine in a very small antimicrobial peptide denoted Aurein 1.2, are involved in its initial binding to the membrane and act as anchors^39^.

Bearing in mind that the FK-1000 fraction is a lyophilized product composed of alanine sequences, phenylalanine, tyrosine and monosaccharides, and that such molecules can exert antimicrobial activities^36–41^, is reasonable to expect that the FK-1000 fraction also exhibits antimicrobial activity.

One important property encountered in the FK-1000 fraction is its potential to be used in combination therapy, which was demonstrated by its ability to induce a fivefold-increase in the action of the aminoglycoside streptomycin against *P. aeruginosa*. This result corroborates those from other studies that demonstrated that exogenous alanine and/or glucose restores the susceptibility to kanamycin in kanamycin-resistant bacteria by increasing antibiotic uptake^40,42,43^.

The potential of the FK-1000 fraction to be used in combination therapy is especially relevant in the case of Gram-negative bacteria, where the incidence of multidrug resistant organisms is high, and it is expected that out of the 252 antimicrobial agents that are being developed, only two to five will become available in 10 years^44^. In addition, outbreaks of Extended Spectrum Beta-lactamases (ESBL) *P. aeruginosa*, such as the ATCC strain utilized in this study, have been described and occurrences are expected to rise in the future^45^.

Finally, the FK-1000 fraction is not toxic to human epithelial cells at concentrations higher than those necessary to induce a bactericidal effect.

It is important to emphasize that despite the fact that the FK-1000 fraction is a mixture of different components whose non-antimicrobial RP HPLC sub-fraction 2 could not even be identified by proton NMR analysis, the standard operating procedure (SOP) to obtain the FK-1000 fraction was standardized and its reproducibility was validated by determining its molecular signature.

The effects observed in the studies reported here have been obtained with relatively high concentrations of the fraction identified, therefore, it is expected that further purification will result in isolation of individual components which are active at much lower concentrations, yielding molecules which have potential for commercialization as new antibiotic treatments. In summary, this study demonstrates that some samples of milk kefir grains can produce a class of molecule different from bacteriocins, ethanol, organic acids and hydrogen peroxide that can also exert antimicrobial activity.

## Supplementary information

Supplementary Figure S1.
