# Supplementary material for: A new class of antimicrobial molecules derived from kefir, effective against Pseudomonas aeruginosa and methicillin resistant Staphylococcus aureus (MRSA) strains
Source: Sci Rep. 2020 Oct 15;10:17434. doi: 10.1038/s41598-020-73651-7 (PMC7566650; doi:10.1038/s41598-020-73651-7)
Supplement: Supplementary file 1 — Supplementary Figure S1. [file 41598_2020_73651_MOESM1_ESM.pdf]

**Supplementary information.**

# A new class of antimicrobial molecules derived from kefir, effective against *Pseudomonas aeruginosa* and Methicillin Resistant *Staphylococcus aureus* (MRSA) strains.

Vaniky Duarte Marques[1], Marcia Regina Franzolin[1], Sabri Saeed Sanabani[2], Hugo Vigerelli[3], Roxane Maria Fontes Piazza[1], Daniel Carvalho Pimenta[3], Tiago Venâncio[4], Irys Viana Neves[1], Herbert Guimarães de Sousa Silva[1], Daniella dos Santos Courrol[1], Lucia Mendonça Previato[5], José Osvaldo Previato[5], Soraia Attie Calil Jorge[6], Marta de Oliveira Domingos[1]*.

[1]Laboratório de Bacteriologia, Instituto Butantan, São Paulo, SP, Brasil; [2] Instituto de Medicina Tropical, Faculdade de Medicina da Universidade de São Paulo, São Paulo, SP, Brasil; [3]Laboratório de Bioquímica e Biofísica, Instituto Butantan, São Paulo, SP, Brasil; [4]Laboratório de Ressonância Magnética Nuclear, Departamento de Química, Universidade Federal de São Carlos, São Carlos, SP, Brasil; [5]Laboratório de Glicobiologia, Instituto de Biofísica Carlos Chagas Filho, Universidade Federal do Rio de Janeiro, Rio de Janeiro, RJ, Brasil; [6] Laboratório de Imunologia Viral, Instituto Butantan, São Paulo, SP, Brasil.

*Corresponding author: Marta de Oliveira Domingos, phone +55 11 26279708 email: marta.domingos@butantan.gov.br

**Supplementary figure**

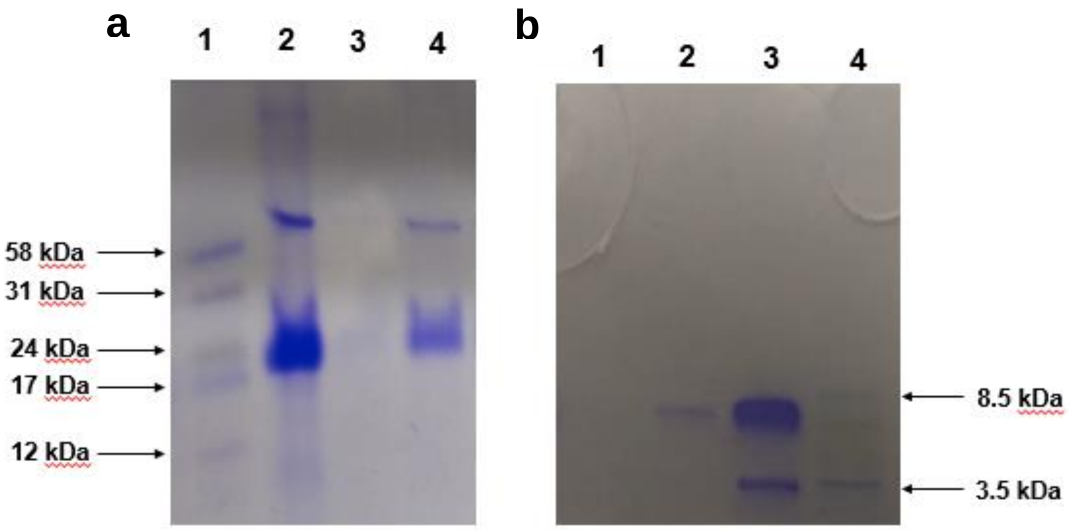


**Supplementary Figure S1 –** The figure shows the full original uncropped images for the Tris-Tricine gels of Fig. 4a,b displayed in the text and results**.**

**a - Tris-Tricine 10 % gel** - **1** Molecular weight, **2** Kefir serum, **3** Kefir molecular fraction with mass > than 10 kDa (Diluted 1/10), **4** Kefir molecular fraction with mass > than 10 kDa (Diluted 1/2); **b** - **Tris-Tricine gel 16 %** - **1** FK-1000 Fraction, **2** Fraction with mass between 1 and 10 kDa (Diluted 1/10); **3** Fraction with mass between 1 and 10 kDa (Diluted 1/2); **4** Molecular weight.
